# Association Between Cognitive and Place-of-Care Trajectories in the Last Three Years of Life

**DOI:** 10.1093/geroni/igaf122.4290

**Published:** 2025-12-31

**Authors:** Haiqun Lin, Olga Jarrín, Ayana April-Sanders, Weiyi Xia, Anum Zafar

**Affiliations:** Rutgers, The State University of New Jersey, Newark, New Jersey, United States; Rutgers, The State University of New Jersey, New Brunswick, New Jersey, United States; Rutgers, The State University of New Jersey, piscataway, New Jersey, United States; Rutgers, The State University of New Jersey, piscataway, New Jersey, United States; Rutgers, The State University of New Jersey, New Brunswick, New Jersey, United States

## Abstract

We examined how patterns of place-of-care align with distinct cognitive function trajectories in the last three years of life. We used a 10% random sample of 181,463 Medicare beneficiaries aged 50 or older who died in 2019. Cognitive function was classified, based on assessment and claims Data: no impairment, memory loss, mild impairment, or dementia. Place-of-care was categorized quarterly for the last three years of life as: (1) home without skilled care; (2) home with skilled care (e.g., home health care or home hospice); or (3) facility-based care, including hospitals or skilled nursing facilities. Group-based trajectory models identified 10 distinct cognitive function trajectories and 13 place-of-care trajectories. One-third of decedents (36%) followed a no-impairment trajectory, spending most of their final three years at home. Two trajectories (7.5%) showed a gradual decline from memory loss to impairment near death, while one (12.1%) exhibited persistent severe impairment and dementia. Beneficiaries in these three declining and impaired trajectories (19.6%) were more likely to use sustained facility-based or skilled home care. These groups also had the highest proportions of females, dual Medicare–Medicaid enrollees, and individuals with depression, psychosis, or stroke. Findings suggest that distinct cognitive decline patterns are associated with predictable place-of-care trajectories and socio-clinical profiles. Targeting care models, such as proactive home-based services and enhanced caregiver support, could reduce reliance on facility-based care and better align end-of-life care with patient and family preferences for high-risk cognitive-trajectory groups. These findings can inform emerging dementia care models and national policy initiatives at improving end-of-life care.

